# Receipt and effectiveness of influenza vaccination reminders for adults, 2011‐2012 season, United States

**DOI:** 10.1111/irv.12568

**Published:** 2018-06-29

**Authors:** Katharine M. Benedict, Tammy A. Santibanez, Katherine E. Kahn, Laura J. Pabst, Carolyn B. Bridges, Erin D. Kennedy

**Affiliations:** ^1^ IHRC, Inc. Atlanta GA USA; ^2^ Centers for Disease Control and Prevention (CDC), National Center for Immunization and Respiratory Diseases (NCIRD), Immunization Services Division (ISD) Atlanta GA USA; ^3^ Leidos, Inc. Atlanta GA USA

**Keywords:** adult, human, influenza, logistic models, population‐based interventions, practice guidelines, preventive health services, vaccination

## Abstract

**Background:**

Reminders for influenza vaccination improve influenza vaccination coverage. The purpose of this study was to describe the receipt of reminders for influenza vaccination during the 2011‐12 influenza season among US adults.

**Methods:**

We analyzed data from the March 2012 National Flu Survey (NFS), a random digit dial telephone survey of adults in the United States. Relative to July 1, 2011, respondents were asked whether they received a reminder for influenza vaccination and the source and type of reminder they received. The association between reminder receipt and demographic variables, and the association between influenza vaccination coverage and receipt of reminders were also examined.

**Results:**

Of adults interviewed, 17.2% reported receiving a reminder since July 1, 2011. More than half (65.2%) of the reminders were sent by doctor offices. Hispanics and non‐Hispanic blacks were more likely than non‐Hispanic whites to report receiving a reminder. Adults who reported having a usual healthcare provider, health insurance, or a high‐risk condition were more likely to report receiving reminders than the respective reference group. Adults reporting receipt of reminders were 1.15 times more likely (adjusted prevalence ratio, 95% CI: 1.06‐1.25) to report being vaccinated for influenza than adults reporting not receiving reminders.

**Conclusions:**

Differences exist in receipt of influenza vaccination reminders among adults. Reminders are important tools to improve adult influenza vaccination coverage. Greater use of reminders may lead to higher rates of adult influenza vaccination coverage and reductions in influenza‐related morbidity.

## 1 INTRODUCTION

Millions of influenza‐related illnesses and outpatient medical visits, and between 140 000‐710 000 influenza‐related hospitalizations and 12 000‐56 600 influenza‐related deaths occur annually in the United States.^1^ Annual influenza vaccination is the most effective method for preventing influenza.^2^, ^3^ Since the 2010‐11 influenza season, annual influenza vaccination has been recommended by the Advisory Committee on Immunization Practices (ACIP) for all persons ≥6 months of age.^2^, ^3^ Prior to this recommendation, the ACIP recommendations focused on children 6 months‐18 years, adults ≥50 years, and persons at high‐risk for complications from influenza and their close contacts; recommendations did not include adults 19‐49 years without a high‐risk condition.^4^ Despite the inclusion of all adults in the universal recommendation, influenza vaccination coverage among adults remains low.^5^, ^6^ Efforts to increase annual influenza vaccination coverage among adults are needed.

Influenza vaccination reminders have been documented to improve influenza vaccination rates and are recommended by ACIP to be implemented by providers.^2^, ^7^, ^8^ Only 25% of primary care physicians issued reminders in the 2010‐11 influenza season,^9^ and adults reported a similar rate (23.2%) during the 2008‐09 vaccination season.^10^ Delivery methods for reminders have included letters, postcards, phone calls, and text messages to mobile devices.^11^, ^12^, ^13^ Reminders can be automatically generated through electronic medical records, pharmacy data systems, or immunization information systems (IIS).^2^, ^8^, ^14^, ^15^, ^16^

This study was designed to describe the receipt of reminders by adults during the 2011‐12 influenza season and differences by sex, age, education, race/ethnicity, having a usual healthcare provider (HCP), health insurance status, and high‐risk condition. Additionally, associations between influenza vaccination coverage and reminders of influenza vaccination were investigated.

## 2 METHODS

### 2.1 Data source

We analyzed data on respondents aged 18 years and older from the March 2012 National Flu Survey (NFS). Details regarding the NFS conducted during the 2011‐12 influenza season in the United States have been previously reported.^17^, ^18^ The NFS was a random digit dial telephone survey that collected data from two sample frames: one landline and one cellular telephone. Households were screened into the survey based on the presence of a household member aged 18 years or older. Cellular telephone respondents were screened into the survey if they reported that they do not maintain a landline telephone in their household or they maintain a landline but make and receive most of their calls on a cellular telephone.

### 2.2 Survey instrument

Interviews were conducted from March 1‐29, 2012. Relative to July 1, 2011, adults were asked whether they received an influenza vaccination, whether they received any reminder for influenza vaccination, and how many times they visited a HCP. Adults who reported they received a reminder were asked who sent the reminder and how the reminder was sent. Respondents were also asked whether they had a HCP that they usually go to for preventive care (usual HCP). These questions were asked as follows: (1) “Since July 1st, 2011 have you had a flu vaccination? It could have been a shot or a spray, drop, or mist in the nose.” (2) “Since July 1st, 2011, did your doctor or other health professional remind you in some way by mail, email, phone call, or text message that you should get a flu vaccination this flu season? Posted signs, newsletters, pamphlets, or television and radio ads should not be considered a reminder.” (3) “How was the reminder communicated to you? (choices: Mail, Phone Call, Email, Text Message, Other, Don't Know, Refused)” (4) “Who sent you this reminder? (choices: Doctor's office, Health clinic, Insurance company, Pharmacy, Other, Don't Know, Refused)” (5) “Is there a place you usually go when you need routine or preventive medical care, such as a physical exam or check‐up?” (6) “How many times since July 1st have you visited a doctor or other health professional about your own health at a doctor's office, hospital, clinic, or some other place?”.^19^ Respondents were considered to have received a reminder if they answered “yes” to question 2) above.

Adults who reported visiting a HCP at least once since July 1, 2011 were asked whether they received a recommendation for influenza vaccination. Those reporting they received a recommendation were asked whether they received an offer to be vaccinated. Participants were asked, “At one or more of these visits, did your doctor or other health care professional recommend that you should get a flu vaccination, should not get a flu vaccination, or did not give a recommendation either way?” This variable was dichotomized into “recommendation” or “no recommendation.” Recommendation to get an influenza vaccination was reported by 5364 respondents. No recommendation included those visiting a HCP who were given a recommendation to *not* get an influenza vaccination (n = 307), those visiting a HCP who were not given a recommendation either way (n = 5765), and adults who did not visit a HCP and thus did not get a recommendation (n = 3427). The question regarding offer of influenza vaccination was “During your visits to the doctor or other health professional, did your doctor or other health professional offer the flu vaccination to you?”

Demographic questions asked of adults included age, sex, race/ethnicity, and level of education. Participants were asked whether they had health insurance or a medical condition which placed them at an increased risk for influenza‐related complications (high‐risk condition). To classify someone as having a high‐risk condition, participants were asked whether they had ever been told by a doctor or other health professional that they had asthma, diabetes, heart disease, a lung condition other than asthma, a kidney condition, obesity, sickle cell anemia or other anemia, a neurological or neuromuscular condition, a liver condition, or a weakened immune system caused by chronic illness or by medications taken for chronic illness. Participants responding that they had been told they had one of these conditions and who currently had the conditions were considered to have a high‐risk condition.

### 2.3 Statistical methods

The percentage of adults who received a reminder for influenza vaccination since July 1, 2011 was calculated overall and by demographic and other characteristics. Wald chi‐square and pair‐wise comparison t tests were conducted to test associations. Among adults who received a reminder, the percentages of who sent the reminders and how the reminders were communicated were also calculated. Respondents who refused to answer a specific question or who answered “don't know” were excluded from analyses.

Bivariate analyses were conducted to investigate associations between the dependent variables (receipt of reminders or influenza vaccination) and each independent variable. Two multivariable models were analyzed with receipt of reminder for influenza vaccination and influenza vaccination as the dependent variables. Independent variables were sex, age (18‐49, 50‐64, or 65+ years), education (<12 years, 12 years, some college, college graduate), race/ethnicity (Hispanic, black non‐Hispanic, white non‐Hispanic, Asian non‐Hispanic, other, or multiracial non‐Hispanic), having a usual HCP (yes, no), health insurance status (yes, no), and high‐risk condition (yes, no). All variables mentioned above were maintained in the adjusted model in Table 2. The model with influenza vaccination as the dependent variable included receipt of reminder as an independent variable and two additional independent variables: number of HCP visits (0 visits, 1 visit, 2‐3 visits, 4‐9 visits, or ≥10 visits) and recommendation and offer for influenza vaccination (recommendation and offer, recommendation but no offer, or no recommendation (including those with no doctor visit since July 1). The “no recommendation” category included respondents who reported not visiting a HCP since July 1, 2011. However, adults who did not have information about influenza vaccination (0.3%, n = 41) were excluded from the model used to explore the relationship between receipt of a reminder and influenza vaccination.

Unadjusted and adjusted prevalence estimates were reported with 95% confidence intervals (95% CI) based on predicted marginals. Similarly, adjusted prevalence ratios (APR) were reported with 95% CIs. All differences noted in the results were statistically significant at a *P‐*value < .05. Analyses were conducted using SAS release 9.3 (SAS Inc. Cary, NC) and SUDAAN release 11.0.0 (Research Triangle Park, NC, http://sudaansupport.rti.org/sudaan/page.cfm/About_SUDAAN) statistical software to take into account the complex survey design. All estimates were weighted based upon probability of selection of the telephone number, adjustments for non‐response at the household level and screening stage, probability of selecting the adult of interest in the household, person non‐response, and a ratio adjustment to population controls (age, sex, race/ethnicity, and geographic area). The NFS was reviewed and approved by the CDC National Center for Health Statistics Ethics Review Board, as part of the National Immunization Survey family of surveys.

## 3 RESULTS

### 3.1 Study population

The Council of American Survey Research Organizations (CASRO) response rate was 31.4% for landline and 18.3% for cellular telephones^19^; the sample included 15 630 adults. Of the 15 630 adult respondents, 515 (3.29%) were missing a response for the reminder question, limiting our sample size to 15 115 respondents.

### 3.2 Receipt of reminders

Among study participants, 17.2% of adults reported receiving reminders for influenza vaccination during the 2011‐12 season (Table 1). Hispanics (19.6%) and non‐Hispanic blacks (24.8%) were more likely to report receiving reminders than non‐Hispanic whites (14.4%).

**Table 1:** Characteristics of adult respondents aged 18 y and older overall and among those who received a reminder for influenza vaccination since July 1, 2011, United States, March 2012 National Flu Survey

Characteristics	Total		Received a Reminder^h^
	n^e^	Weighted^f^ % (95% CI^g^)	Weighted % (95% CI)
Total	‐	‐	17.2 (16.1‐18.4)
Sex			
Female	7473	50.2 (48.7‐51.7)	18.3 (16.7‐20.0)
Male	7642	49.8 (48.3‐51.3)	16.1 (14.6‐17.7)
Age			
18‐49 y	5776	58.6 (57.3‐60.0)	17.4 (15.8‐19.2)
50‐64 y	4605	24.6 (23.5‐25.8)	16.2 (14.4‐18.1)
65+ y	4734	16.7 (15.9‐17.6)	18.0 (16.3‐19.8)
Education			
<12 y	1194	10.0 (9.1‐11.1)	19.7 (15.7‐24.4)
12 y	2680	22.6 (21.2‐24.0)	15.8 (13.5‐18.3)
Some college	3614	29.2 (27.7‐30.7)	16.8 (14.6‐19.4)
College graduate	6169	38.3 (36.8‐39.8)	17.2 (15.6‐18.9)
Race/ethnicity			
Hispanic	1586	13.9 (12.8‐15.2)	19.6 (16.0‐23.8)^c^
Black, non‐Hispanic	1734	12.1 (11.0‐13.2)	24.8 (20.9‐29.2)c,d
White, non‐Hispanic	10687	67.3 (65.8‐68.8)	15.4 (14.2‐16.6)a,b
Asian, non‐Hispanic	660	4.3 (3.8‐4.9)	15.9 (12.4‐20.2)^b^
Other or Multiracial, non‐Hispanic	448	2.3 (1.9‐2.9)	18.8 (13.4‐25.7)
Usual HCP^i^			
Yes	13669	87.9 (86.8‐88.9)	18.6 (17.4‐19.9)^b^
No	1425	12.1 (11.1‐13.2)	6.9 (5.2‐9.1)^a^
Health Insurance^j^			
Yes	12158	82.5 (81.0‐83.8)	18.1 (16.9‐19.4)^b^
No	1548	17.5 (16.2‐19.0)	11.8 (9.5‐14.7)^a^
High‐Risk Condition^k^			
Yes	4875	29.2 (27.9‐30.6)	20.7 (18.6‐22.9)^b^
No	9159	70.8 (69.4‐72.1)	15.7 (14.3‐17.1)^a^

^a,b,c,d^The presence or absence of superscripted letters denotes whether that estimate was significantly different at *P *<* *.05 from another row, and denotes which row it differed from (a, b, c, d), based on pair‐wise comparison *t* test. For example, the percentage of Hispanics who reported receiving reminders (19.6%) was significantly different from the percentage of non‐Hispanic whites who reported receiving reminders (15.4%).

^e^Unweighted sample size.

^f^Weighting based on two sample frames (landline and cell phone) subdivided into two strata: an oversampling area and a non‐oversampling area, to achieve higher proportional representation among three minority race/ethnicity groups—Hispanic, non‐Hispanic black, and non‐Hispanic Asian. Oversampling among landline telephones was performed at the county level. Oversampling for cell phone was performed at the state level.

^g^95% confidence intervals; all percentages and CIs are based on weighted analysis of data using SUDAAN.

^h^”Since July 1, 2011, did your doctor or other health professional remind you some way by mail, email, phone call, or text message to get a flu vaccination? Posted signs, newsletters, pamphlets, or television and radio ads were not considered a reminder.”

^i^”Is there a place you usually go when you need routine or preventive medical care, such as a physical exam or check‐up?”

^j^”Do you have any kind of health care coverage, including health insurance, prepaid plans such as HMOs, or government plans such as Medicare?”

^k^To classify someone as having a high‐risk condition, participants were asked a series of related questions. First, participants were asked whether a doctor, nurse, or other health professional had ever said the survey participant has asthma, diabetes, heart disease, or any of the following list of health conditions: a lung condition other than asthma, a kidney condition, obesity, sickle cell anemia or other anemia, a neurological or neuromuscular condition, a liver condition, or a weakened immune system caused by chronic illness or by medications taken for chronic illness. Participants answering “yes” that they had ever been told they had one of these conditions were then asked whether they still have asthma, diabetes, heart disease, or any one of these additional conditions. Anyone indicating that they still had asthma, diabetes, heart disease, or any one of the additionally listed conditions was considered to have a high‐risk condition in this analysis.

HCP, healthcare professional.

Participants who reported having a usual HCP (18.6%), health insurance (18.1%), or a high‐risk condition (20.7%) were more likely to report receiving reminders compared to the respective reference groups.

**Figure 1:**
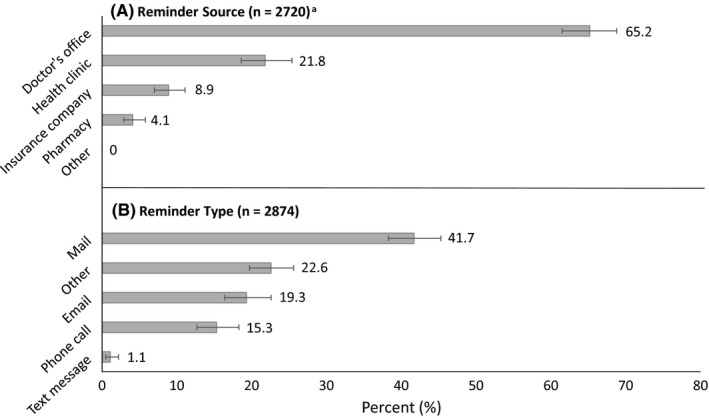
Reported source of reminder (A) for influenza vaccination and the type of reminder received (B), United States, March 2012 National Flu Survey (NFS). ^a^Missing responses, refusals, and responses of “don't know” for source (n = 201, 6.9%) and type (n = 46, 1.6%) of reminder were excluded from analyses

Reminders for influenza vaccination were most often reported to be sent by a doctor's office (65.2%), followed by a health clinic (21.8%), an insurance company (8.9%), and a pharmacy (4.1%) (Figure 1). The reminder was reported to be communicated by mail (41.7%), email (19.3%), phone call (15.3), text message (1.1%), or some other source (22.6%).

Having a usual HCP (APR = 2.56, 95% CI: 1.85‐3.54) was the strongest predictor of reporting receipt of a reminder for influenza vaccination when controlling for sex, age category, race/ethnicity, education, health insurance, and high‐risk condition (Table 2). Hispanics (APR = 1.50, 95% CI: 1.20‐1.88) and non‐Hispanic blacks (APR = 1.70, 95% CI: 1.40‐2.05) were more likely to report receiving reminders than non‐Hispanic whites when controlling for the other variables. Adults who reported having health insurance (APR = 1.38, 95% CI: 1.07‐1.78) or a high‐risk condition (APR = 1.24, 95% CI: 1.08‐1.44) were more likely to report receiving reminders for influenza vaccination compared to the respective reference group.

**Table 2:** Association between demographic characteristics and receipt of reminders^a^ for influenza vaccination, since July 1, 2011, March 2012 National Flu Survey, United States

Characteristic	Adjusted^b^	
	Reminder prevalence^c^	Reminder prevalence ratio^d^ (95% CI^e^)
Sex		
Female	17.4 (15.8‐19.2)	1.04 (0.91‐1.20)
Male	16.7 (15.2‐18.4)	Ref^f^
Age		
18‐49 y	17.9 (16.2‐19.9)	Ref
50‐64 y	15.7 (13.9‐17.6)	0.87 (0.75‐1.02)
65+ y	16.4 (14.7‐18.4)	0.92 (0.78‐1.07)
Education		
<12 y	18.4 (14.6‐23.0)	Ref
12 y	16.2 (13.9‐18.9)	0.88 (0.67‐1.15)
Some college	16.8 (14.7‐19.2)	0.91 (0.70‐1.19)
College graduate	17.4 (15.7‐19.2)	0.94 (0.73‐1.21)
Race/ethnicity		
Hispanic	21.9 (17.9‐26.6)	**1.50 (1.20‐1.88)** g
Black, non‐Hispanic	24.7 (20.9‐29.0)	**1.70 (1.40‐2.05)**
White, non‐Hispanic	14.6 (13.3‐16.0)	Ref
Asian, non‐Hispanic	16.9 (13.1‐21.6)	1.16 (0.89‐1.52)
Other or multiracial, non‐Hispanic	19.0 (13.4‐26.2)	1.30 (0.92‐1.85)
Usual HCP^h^		
Yes	18.3 (17.0‐19.6)	**2.56 (1.85‐3.54)**
No	7.1 (5.2‐9.7)	Ref
Health insurance^i^		
Yes	17.9 (16.5‐19.3)	**1.38 (1.07‐1.78)**
No	13.0 (10.2‐16.3)	Ref
High‐risk condition^j^		
Yes	19.7 (17.6‐22.1)	**1.24 (1.08‐1.44)**
No	15.9 (14.5‐17.3)	Ref

Bold values indicate statistically significant odds ratios.

a ”Since July 1, 2011 did the survey participant's doctor or other health professional remind the survey participant in some way by mail, email, phone call, or text message to get a flu vaccination?” Posted signs, newsletters, pamphlets, or television and radio ads were not considered a reminder?

b Adjusted for sex, age, education, race/ethnicity, usual HCP, health insurance, and high‐risk condition (n = 13 472).

c The predicted marginal model was used to estimate recommendation prevalence.

d Prevalence ratio interpreted as the odds of report of recommendation given the characteristic for the exposure variable compared to the exposure variable reference group.

e 95% confidence intervals.

f Reference group.

g Bolded prevalence ratios and 95% CI indicate statistical significance, *P *<* *.05.

h ”Is there a place you usually go when you need routine or preventive medical care, such as a physical exam or check‐up?”

i ”Do you have any kind of health care coverage, including health insurance, prepaid plans such as HMOs, or government plans such as Medicare?”

j To classify someone as having a high‐risk condition, participants were asked a series of related questions. First, participants were asked whether a doctor, nurse, or other health professional had ever said the survey participant has asthma, diabetes, heart disease, or any of the following list of health conditions: a lung condition other than asthma, a kidney condition, obesity, sickle cell anemia or other anemia, a neurological or neuromuscular condition, a liver condition, or a weakened immune system caused by chronic illness or by medications taken for chronic illness. Participants answering “yes” that they had ever been told they had one of these conditions were then asked whether they still have asthma, diabetes, heart disease, or any one of these additional conditions. Anyone indicating that they still had asthma, diabetes, heart disease, or any one of the additionally listed conditions was considered to have a high‐risk condition in this analysis.

HCP, healthcare professional.

### 3.3 Association between receipt of reminders and vaccination coverage

Among adult respondents, 45.3% (95% CI: 43.8‐46.8) reported receiving an influenza vaccination since July 1, 2011. During the 2011‐12 influenza season, a higher percentage of adults who reported receipt of a reminder for influenza vaccination were vaccinated (57%, 95% CI: 53.4‐60.5) compared to adults who did not report receipt of a reminder (42.8%, 95% CI: 41.2‐44.5) (*P *<* *.0001). Based on the multivariable model, adults who reported receiving a reminder for influenza vaccination were more likely to report being vaccinated for influenza than adults who did not receive a reminder (APR 1.15, 95% CI: 1.06‐1.25, *P *<* *.05), controlling for all independent variables.

## 4 DISCUSSION

About 17% of adults reported receiving reminders during the 2011‐12 influenza season. The rate of receiving reminders for influenza vaccination may have decreased from the 23% estimate reported from the 2008‐09 vaccination season.^10^ Of all the variables investigated in our study, having a usual HCP has the strongest association with reported receipt of a reminder, and most of the reminders were reported to have been sent by doctors’ offices. In addition, report of receipt of an influenza vaccination reminder during the 2011‐12 influenza season was associated with receipt of influenza vaccination.

Non‐Hispanic blacks and Hispanics were more likely to report receiving reminders than non‐Hispanic whites. This could be due to differences in the types of healthcare facilities that these groups typically visit for their influenza vaccinations. Studies show that non‐Hispanic blacks and Hispanics are more likely to be vaccinated in medical settings compared to non‐Hispanic whites.^20^ Our study found that the majority of reminders received were from doctor's offices and few reminders came from non‐medical settings such as pharmacies. Therefore, if medical settings developed their reminders based on previous season vaccination, those non‐Hispanic blacks and Hispanics being vaccinated in medical settings would be more likely than non‐Hispanic whites to receive reminders. Additionally, during the 2011‐12 season, non‐medical settings were more commonly reported as place of adult influenza vaccination compared to the 2010‐11 season; therefore, these settings should consider sending reminders to all of their patients.^20^ Additionally, other possible sources of reminders for influenza vaccination (ie, insurance companies) may reach healthy adults who may not routinely see providers during an influenza season.

Although we found non‐Hispanic black and Hispanic adults to be more likely than non‐Hispanic white adults to receive a reminder for influenza vaccination, vaccination coverage among these adults is lower than non‐Hispanic whites.^5^, ^6^ Reminders may not be as effective for these groups, perhaps due to different knowledge and attitudes about influenza vaccination or how the reminders are communicated. Providers should not only send reminders to all patients but also ensure that the reminders they send are culturally sensitive.^21^ Exploring why reminders might not be as effective in these groups in future studies would be beneficial. This information could provide guidance on the most influential language, formats, and sources of reminders for influenza vaccination. Previous research has indicated a variety of other factors that contribute to racial/ethnic differences in adult vaccination rates, including patient, provider, and system factors.^22^, ^23^, ^24^, ^25^

Prior to the universal influenza vaccination recommendation, individuals with high‐risk conditions were a priority group for annual influenza vaccination.^2^, ^4^ In 2000, healthcare practices able to generate lists of elderly patients and patients with chronic illness were more likely to send reminders about the need for influenza vaccination compared to other practices.^26^ Our finding that adults with high‐risk conditions were more likely to report receiving reminders could reflect a continuation of this practice. Further, individuals with chronic illnesses have more HCP visits than healthy people and may be more likely to be included in system queries to produce reminders.^27^ Current provider practices regarding reminders for influenza vaccination of adults should include all patients.

More than 20% of the adults receiving reminders for influenza vaccination in our study reported the reminder was received in some manner “other” than mail, phone, email, or text. “Other” might represent confusion between reminders and recommendations for influenza vaccination given during HCP visits. Although clear distinctions exist between the definitions of reminders and recommendations, these distinctions may not have been clear for survey participants. Analysis of receipt of recommendations for influenza vaccination from NFS has been reported separately.^28^ Respondents may also have received multiple types of reminders and selected “other” due to their inability to select multiple methods in the survey.

A previous study found that employers were the most important source of reminders.^10^ However, employer was not an option for reminder source in our questionnaire. Although no respondents answered “other” regarding the source of the reminder they received, if employer had been a potential answer, perhaps respondents would have remembered the reminder they received from these sources.

Studies have shown that IIS is an effective source of reminder/recall messages for increasing vaccination rates in pediatric populations^29^, ^30^, ^31^ and the Community Preventive Services Task Force recommends use of IIS.^16^ Barriers to sending reminders for immunization include resource constraints and concerns about access to completed immunization histories.^32^, ^33^ IIS can address these barriers as they consolidate vaccination histories across vaccination providers to provide the most complete immunization history for a patient, and reminder messages can be generated centrally by health departments to reduce resource burdens on providers. The Standards for Adult Immunization Practice calls for immunizing providers to document receipt of vaccination in IIS and for health departments to increase IIS access and use by providers of adults.^34^ Adult participation in IIS was low in 2012, with only 25% participating in an IIS.^35^ As these standards are implemented, IIS may achieve higher rates of adult participation and become even more effective in improving adult influenza vaccination rates.

Several barriers to HCP use of reminders have been identified. One barrier is a lack of an electronic health record (EHR) that could generate reminders. With implementation and adoption of meaningful use of EHR technology, more HCP will likely have EHRs with capacities to generate reminders.^36^ However, a HCP survey in 2010 showed that 55% of HCP issued reminders for check‐ups and well‐care visits but only 40% issued influenza vaccination reminders, suggesting that even when reminder systems exist, they are not used for influenza vaccination.^9^ A published systematic review of the literature found that reminders increased vaccination rates in all types of primary care settings and that all types of reminders were associated with increased vaccination rates.^7^ As most studies focus on specific practice types, HCP may be reluctant to implement reminders if they perceive the findings do not apply to their own practice. Further, HCP may be reluctant because specific recommendations regarding how and how often a reminder should be sent have not been made.

This study has several limitations due to the design and rapid collection of the information. First, all data relied upon self‐report, including vaccination status that was not validated with medical records, and are subject to recall bias. Second, vaccinated individuals may have been more likely to remember receipt of vaccination reminders. Third, the CASRO response rate was low and non‐response bias may remain even after weighting adjustments. Fourth, households were excluded that did not have telephone service or that did not respond to early call attempts which could result in non‐response bias. Fifth, selection bias could occur if respondents had particularly strong feelings for or against influenza vaccination. Additionally, interaction terms were not included in the models; therefore, the APR for effect of reminder may be underestimated.

In conclusion, receipt of reminders for influenza vaccination was infrequently reported to be received by adults during the 2011‐12 influenza season; however, receipt of reminders was associated with adult influenza vaccination. Reminders of influenza vaccination are important tools in the efforts to improve adult influenza vaccination coverage, and full integration of reminders into all healthcare systems could help improve factors that potentially contribute to current differences in receipt of reminders. To assist with efforts to increase influenza vaccination coverage among adults, all adults should be reminded to get vaccinated at the beginning of each season regardless of whether they have a scheduled provider visit. A variety of methods (ie, postcards, phone calls, text messages, and emails) to communicate reminders of influenza vaccination can be utilized to reach adults of all ages.

## DISCLAIMER

The findings and conclusions in this report are those of the authors and do not necessarily represent the official position of the Centers for Disease Control and Prevention.

## References

[1] Centers for Disease Control and Prevention . Estimated influenza illnesses, medical visits, hospitalizations, and deaths averted by vaccination in the United States. https://www.cdc.gov/flu/about/disease/2015-16.htm. Accessed 18 December 2017.

[2] Fiore AE , Uyeki TM , Broder K , et al. Prevention and control of influenza with vaccines: recommendations of the Advisory Committee on Immunization Practices (ACIP), 2010. MMWR Recomm Rep. 2010;59:1‐62. 20689501

[3] Grohskopf LA , Shay DK , Shimabukura TT , et al. Prevention and control of seasonal influenza with vaccines. Recommendations of the Advisory Committee on Immunization Practices—United States, 2013–2014. MMWR Recomm Rep. 2013;62:1‐43. 24048214

[4] Fiore AE , Shay DK , Broder K , et al. Prevention and control of influenza with vaccines: recommendations of the Advisory Committee on Immunization Practices (ACIP), 2009. MMWR Recomm Rep. 2010;58:1‐52. 19644442

[5] Lu PJ , Singleton JA , Euler GL , Williams WW , Bridges CB . Seasonal influenza vaccination coverage among adult populations in the United States, 2005‐2011. Am J Epidemiol. 2013;178:1478‐1487. 24008912 10.1093/aje/kwt158 PMC5824626

[6] Santibanez TA , O'Halloran A , Yusheng Z , et al. Flu vaccination coverage, United States, 2013‐14 influenza season. http://www.cdc.gov/flu/fluvaxview/coverage-1314estimates.htm. Accessed September 18, 2014.

[7] Jacobson Vann JC , Szilagyi P . Patient reminder and recall systems to improve immunization rates. Cochrane Database of Syst Rev. 2005:CD003941. 10.1002/14651858.CD003941.pub2 16034918 PMC6485483

[8] Grohskopf LA , Olsen SJ , Sokolow LZ , et al. Prevention and control of seasonal influenza with vaccines: recommendations of the national vaccine Advisory Committee on Immunization Practices (ACIP) – United States, 2014‐2015 influenza season. MMWR. 2014;63:691‐697. 25121712 PMC4584910

[9] Maurer J , Harris KM . Issuance of patient reminders for influenza vaccination by US‐based primary care physicians during the first year of universal influenza vaccination recommendations. Am J Public Health. 2014;104:e60‐e62. 10.2105/AJPH.2014.301888 PMC4061990 24825233

[10] Maurer J , Harris KM . The scope and targeting of influenza vaccination reminders among US adults: evidence from a nationally representative survey. Arch Intern Med. 2010;170:390‐392. 20177045 10.1001/archinternmed.2009.520

[11] Anderson KK , Sebaldt RJ , Lohfeld L , et al. Patient views on reminder letters for influenza vaccinations in an older primary care patient population. Can J Public Health. 2008;99:133‐136. 18457289 10.1007/BF03405461 PMC6976018

[12] Szilagyi PG , Adams WG . Text messaging: a new tool for improving preventive services. JAMA. 2012;307:1748‐1749. 22535860 10.1001/jama.2012.524

[13] Stockwell MS , Westhoff C , Karbanda EO , et al. Influenza vaccine text message reminders for urban, low‐income pregnant women: a randomized controlled trial. Am J Public Health. 2014;104:e7‐e12. 10.2105/AJPH.2013.301620 PMC4011110 24354839

[14] Pearson D , Jackson LA , Winkler B , Foss B , Wagener B . Use of an automated pharmacy system and patient registries to recruit HMO enrollees for an influenza campaign. Eff Clin Prac. 1998;2:17‐22. 10346549

[15] Dombowski KJ , Harrington L , Hanauer D , Kennedy A , Clark S . Current and potential use of new technologies for reminder notifications. Clin Pediatr. 2012;51:394‐397. 10.1177/0009922811420715 22333573

[16] The guide to community preventive services: The community guide, what works to promote health. Increasing appropriate vaccination: Immunization Information Systems. http://www.thecommunityguide.org/vaccines/imminfosystems.html. Accessed 16 June 2015.

[17] Kennedy ED , Santibanez TA , Bridges CB , Singleton JA . National mid‐season flu vaccination coverage, National flu survey, United States, 2011‐2012 season. http://www.cdc.gov/flu/fluvaxview/national-flu-survey.htm. Accessed December 5, 2011.

[18] Kennedy ED , Santibanez TA , Bridges CB , Singleton JA . March flu vaccination coverage, United States, 2011‐2012 influenza season. http://www.cdc.gov/flu/fluvaxview/nfs-survey-march2012.htm. Accessed May 16, 2012.

[19] Council of American Survey Research Organizations Task Force on Completion Rates . 1982 On the definition of response rates. CASRO Special Report. Port Jefferson, NY: Council of American Survey Research Organizations. http://c.ymcdn.com/sites/www.casro.org/resource/resmgr/docs/casro_on_definitions_of_resp.pdf?hhSearchTerms=%22response+and+rate%22. Accessed June 19, 2018.

[20] Lu PJ , O'Halloran A , Ding H , Williams WW , Bridges CB , Kennedy ED . National and state‐specific estimates of place of influenza vaccination among adult populations – United States, 2011‐12 influenza season. Vaccine. 2014;32:3198‐3204. 24731815 10.1016/j.vaccine.2014.04.003 PMC5824644

[21] Vlahov D , Coady MH , Ompad DC , Galea S . Strategies for improving influenza immunization rates among hard‐to‐reach populations. J Urban Health. 2007;84:615‐631. 17562184 10.1007/s11524-007-9197-z PMC2219560

[22] Singleton JA , Santibanez TA , Wortley PM . Influenza and pneumococcal vaccination of adults aged ≥ 65: racial/ethnic differences. Am J Prev Med. 2005;29:412‐420. 16376704 10.1016/j.amepre.2005.08.012

[23] Lindley MC , Wortley PM , Winston CA , Bardenheier BH . The role of attitudes in understanding disparities in adult influenza vaccination. Am J Prev Med. 2006;31:281‐285. 16979451 10.1016/j.amepre.2006.06.025

[24] Bach PB , Pham HH , Schrag D , Tate RC , Hargraves JL . Primary care physicians who treat blacks and whites. N Engl J Med. 2004;351:575‐584. 15295050 10.1056/NEJMsa040609

[25] Gemson DH , Elinson J , Messeri P . Differences in physician prevention practice patterns for white and minority patients. J Community Health. 1988;13:53‐64. 3360981 10.1007/BF01321480

[26] Davis MM , McMahon SR , Santoli JM , Schwartz B , Clark SJ . A national survey of physician practices regarding influenza vaccine. J Gen Intern Med. 2002;17:670‐676. 12220362 10.1046/j.1525-1497.2002.11040.x PMC1495108

[27] Anderson G . Chronic care: making the case for ongoing care. Robert Wood Johnson Foundation. 2010 http://www.rwjf.org/pr/product.jsp?id=50968. Accessed June 19, 2018.

[28] Benedict KM , Santibanez TA , Black CL , et al. Recommendations and offers for adult influenza vaccination, 2011‐2012 season, United States. Vaccine. 2017;35:1353‐1361. 27137099 10.1016/j.vaccine.2016.04.061 PMC5689412

[29] Groom H , Hopkins DP , Pabst LJ , et al. Immunization information systems to increase vaccination rates: a community guide systematic review. J Public Health Manag Pract. 2015;21:227‐248. 24912082 10.1097/PHH.0000000000000069

[30] Dombkowski KJ , Costello LE , Harrington LB , Dong S , Kolasa M , Clark SJ . Age‐specific strategies for immunization reminders and recalls: a registry‐based randomized trial. Am J Prev Med. 2014;47:1‐8. 24750973 10.1016/j.amepre.2014.02.009

[31] Dombkowski KJ , Cowan AE , Potter RC , Dong S , Kolasa M , Clark SJ . Statewide pandemic influenza vaccination reminders for children with chronic conditions. Am J Public Health. 2014;104:e39‐e44. 10.2105/AJPH.2013.301662 PMC3910056 24228668

[32] Pereira JA , Quach S , Heidebrecht CL , et al. Barriers to the use of reminder/recall interventions for immunizations: a systematic review. BMC Med Inform Decis Mak. 2012;12:1‐10. 23245381 10.1186/1472-6947-12-145 PMC3541955

[33] Briss PA , Rodewald LE , Himan AR , et al. Reviews of evidence regarding interventions to improve vaccination coverage in children, adolescents, and adults. Am J Prev Med. 2000;18:97‐140. 10.1016/s0749-3797(99)00118-x 10806982

[34] National Vaccine Advisory Committee . Recommendations from the National Vaccine Advisory Committee: standards for Adult Immunization Practice. Public Health Rep. 2014;129:115‐123. 24587544 10.1177/003335491412900203 PMC3904889

[35] CDC . Progress in immunization information systems—United States, 2012. MMWR Morb Mortal Wkly Rep. 2013;62:1005‐1008. 24336133 PMC4585582

[36] HealthyIT.gov. For providers and professionals, EHR incentives and certification: meaningful use definition and objectives. https://www.healthit.gov/providers-professionals/meaningful-use-definition-objectives. Accessed 30 October 2015.

